# Supplementary material for: Combined Use of Presepsin and (1,3)-β-D-glucan as Biomarkers for Diagnosing Candida Sepsis and Monitoring the Effectiveness of Treatment in Critically Ill Patients
Source: J Fungi (Basel). 2022 Mar 17;8(3):308. doi: 10.3390/jof8030308 (PMC8954802; doi:10.3390/jof8030308)
Supplement: Supplementary file 1 [file jof-08-00308-s001.zip › jof-1590979-supplementary.pdf]

Journal of *Fungi*

Supplementary Information: Combined use of presepsin and (1,3)-β-D-glucan as biomarkers for diagnosing *Candida* sepsis and monitoring the effectiveness of treatment in critically ill patients

Radim Dobiáš[1,2,†], Marcela Káňová[3,4,5,*,†], Naděžda Petejová[6,7,8], Štefan Kis Pisti[9], Robert Bocek[10], Eva Krejčí[1,2], Helena Stružková[2], Michaela Cachová[1], Hana Tomášková[11,12], Petr Hamal[13], Vladimír Havlíček[14,15,†] and Milan Raška[16,†]

1 Department of Bacteriology and Mycology, Public Health Institute in Ostrava, 702 00 Ostrava, Czech Republic; radim.dobias@zuova.cz (R.D.); eva.krejci@zuova.cz (E.K.); michaela.cachova@zuova.cz (M.C.)
2 Institute of Laboratory Medicine, Faculty of Medicine, University of Ostrava, 703 00 Ostrava, Czech Republic; helcastruzkova@seznam.cz (H.S.)
3 Department of Anesthesiology and Intensive Care Medicine, University Hospital Ostrava, 708 00 Ostrava, Czech Republic
4 Institute of Physiology and Pathophysiology, Faculty of Medicine, University of Ostrava, 710 00 Ostrava, Czech Republic
5 Department of Intensive Medicine, Emergency Medicine and Forensic Studies, University of Ostrava, 710 00 Ostrava, Czech Republic
6 Department of Internal Medicine, University Hospital Ostrava, 708 00 Ostrava, Czech Republic; petejova@seznam.cz (N.P.)
7 Department of Internal Medicine, University of Ostrava, 703 00 Ostrava, Czech Republic
8 Department of Internal Medicine III – Nephrology, Rheumatology and Endocrinology, Faculty of Medicine and Dentistry, Palacký University Olomouc, 775 15 Olomouc, Czech Republic
9 Department of Anesthesiology and Resuscitation, Ostrava City Hospital, 728 80 Ostrava, Czech Republic; kispisti@seznam.cz (Š.K.P.)
10 Department of Anesthesiology and Resuscitation, Hospital with Polyclinic Havířov, 736 01 Havířov, Czech Republic; robert.bocek@nsphav.cz (R.B.)
11 Center of Health Services, Institute of Public Health in Ostrava, 702 00 Ostrava, Czech Republic; hana.tomaskova@zuova.cz (H.T.)
12 Department of Epidemiology and Public Health, Faculty of Medicine, University of Ostrava, 703 00 Ostrava, Czech Republic,
13 Department of Microbiology, Faculty of Medicine and Dentistry, Palacký University Olomouc, 775 15 Olomouc, Czech Republic; petr.hamal@fnol.cz (P.H.)
14 Institute of Microbiology of the Czech Academy of Sciences, 142 20 Prague, Czech Republic; vlhavlic@biomed.cas.cz (V.H.)
15 Department of Analytical Chemistry, Faculty of Science, Palacký University, 771 46 Olomouc, Czech Republic
16 Department of Immunology, Faculty of Medicine and Dentistry, Palacký University Olomouc, 775 15 Olomouc, Czech Republic; milan.raska@upol.cz (M.R.)
* Correspondence: marcela.kanova@fno.cz (M.K.)
† R.D. and M.K.; V.H. and M.R. contributed equally to this article

* Correspondence: Data on the kinetics of presepsin justifying the choice of sampling window (within 24 h before indexed blood culture) used in the study

S1: Data on the kinetics of presepsin justifying the choice of sampling window (within 24 h before indexed blood culture) used in the study

Presepsin (PSEP) is a collective term for a group of soluble CD14 subtypes (sCD14-ST) that indicate an infectious agent's myelomonocytic activation. PSEP is formed and released into the blood when the cell surface membrane protein mCD14 is cleaved by proteases. In addition to myelomonocytes, hepatocytes and cells in the kidneys and lungs also respond sensitively to the presence of endotoxin by secreting sCD14 [42,43]. PSEP is detectable as early as one hour after endotoxin exposure, with peak levels occurring 3 hours after exposure [44,45]. As such, the PSEP response is faster than that of procalcitonin (PCT), whose levels only begin rising 2–4 hours after exposure [34], and C-reactive protein (CRP), whose levels start growing 12 hours after exposure and reach a maximum at 20-72 hours [46]. The biological half-life of PSEP (five hours) is also shorter than that of PCT (20-24 hours) [44]. These properties make PSEP a valuable early laboratory biomarker of sepsis, and its short biological half-life means that it can be used to evaluate the effectiveness of antibiotic treatments. Both PSEP and PCT can also be used to determine when to discontinue antibiotic therapy: treatment can be halted once their levels fall by more than 80% from the peak value (for PCT, this will typically be below 0.5 μg/mL) [34,44–49].

PSEP is present at very low concentrations in healthy individuals: the reference interval was 55–184 pg/mL, with a slight difference between men and women, in an Italian study [50] on 200 healthy individuals without signs of inflammatory reaction. Its concentration is significantly higher in patients with a systemic inflammatory response [47,51–53]. As with PCT, PSEP levels increase with age [44,47,48] and are also higher in neonates [54].

Multicenter studies have shown that the sensitivity (87.8%) and specificity (81.4%) of PSEP as a biomarker are maximized when using a cut-off value of 600 pg/mL; the use of a lower cut-off increased sensitivity but reduced specificity. PSEP also has a good negative predictive value: levels <200 pg/mL practically rule out systemic infection, and bacterial infection is very unlikely at levels <300 pg/mL. The concentration of PSEP in patients suffering from systemic inflammatory response syndrome of infectious etiology (i.e., a simple infection) is typically lower than in cases of sepsis, where the immune response to an infectious agent leads to the development of organ failure. Its concentrations are highest in patients undergoing septic shock; concentrations ≥ 1,000 pg/mL corresponds to a SOFA score ≥ 8 and have a high predictive value as an indicator of likely sepsis progression to septic shock with multiorgan failure [34,48].

Table S1. Correlations of biomarkers and clinical scores with 28-day mortality reveal significant differences with regard to candidemia (n = 58).
Significant differences in SOFA score, CRP, PCT and PSEP. PSEP is significantly correlated with PCT (Spearman's rho = 0.5811, p = 0.0182).

Death	Test/Score	Centile	Mean	SD	Min	Max	p-value
No	APACHE II	11	14.1	7.6	7	33	0.0549
Yes	APACHE II	18	17.6	6.1	7	26	
No	SOFA	3	5.0	4.3	2	18	0.0117
Yes	SOFA	7.5	8.1	4.7	2	16	
No	BDG	1014	851.9	356.5	106	1307	0.3541
Yes	BDG	1119.5	892.6	419.4	104	1334	
No	CRP	98	107.5	61.6	30	330	0.0034
Yes	CRP	145	173.4	86.4	55	338	
No	PCT	1.4	5.9	16.7	0.24	100	0.0370
Yes	PCT	2.5	4.6	6.0	0.5	24.87	
No	PSEP	1393	2013.0	1507.7	362	5950	0.0048
Yes	PSEP	3236.5	4263.8	4101.2	800	16899	

SOFA Self Organ Failure Assessment score, APACHE II Acute Physiology, and Chronic Health Evaluation II score **PCT p**rocalcitonin, **PSEP p**resepsin, **CRP** C-reactive protein.
Two-sample Wilcoxon rank-sum (Mann-Whitney) test

Table S2. Multivariable logistic regression analysis of the predictive value for mortality of presepsin and procalcitonin in invasive candidiasis patients.
Presepsin is significantly more accurate than PCT.

Test/Deaths	Odds Ratio	Std. Err.	z	P>(z)	95% CI
PCT/1.078656	0.9150811	0.0767836	-1.06	0.290	0.7763115
PSEP/1.001088	1.000608	0.0002445	2.49	0.013	1.000129
Cons/0.386521 8	0.1147259	0.0710987	-3.49	0.000	0.0340525

PCT procalcitonin, PSEP presepsin, CI confidence interval, Cons contras.

Table S3. The distribution of (1,3)-β-D-glucan and presepsin concentrations in patients with different origins of candidemia.

The results of the Kruskal-Wallis equality-of-populations rank test presented in the table below indicate that (1,3)-β-D-glucan has significant value for differentiating between groups 0, 1, and 2 ($P < 0.001$). Conversely, presepsin has no potential for resolving groups 0, 1, and 2 ($P = 0.5375$).

**Patients without catheter colonization**			
**Blood culture Group 1**	**Catheter, PICC/days before/after IBC**	**BDG (pg/mL) IBC**	**PSEP (pg/mL) IBC**
C. parapsilosis	NC	850	837
C. albicans	NC	838	1851
C. albicans	NC	1033	5544
C. tropicalis	NC	1179	1786
C. albicans	NC	1085	2287
C. krusei	NC	1220	1647
C. albicans	NC	1026	5664
C. parapsilosis	NC	1087	1783
G. clavatum	NC	200	3578
C. glabrata	NC	1057	800
C. albicans	NC	1185	16,899

C. albicans	NC	1239	5676
C. tropicalis	NC	399	1325
C. albicans	NC	104	1945
C. glabrata	NC	400	2785
C. krusei	NC	1100	1371
C. albicans	NC	839	714
C. glabrata	NC	593	2468
C. albicans	NC	1077	1381
C. tropicalis	NC	1247	3259
C. albicans	NC	942	3542
C. albicans	NC	843	12,603
C. cerevisiae	NC	1111	1393
C. tropicalis	NC	457	3222
C. albicans	NC	367	3831
C. albicans	NC	1334	3214
C. krusei	NC	1155	740
C. krusei	NC	1140	800
C. krusei	NC	1231	845
C. krusei	NC	1065	1477
C. krusei	NC	1002	876
C. metapsilosis	NC	692	1393
C. guilliermondii	NC	229	1109
C. tropicalis	NC	1014	1380
C. tropicalis	NC	1010	1178
C. tropicalis	NC	1176	843

Catheter-related candidiasis - candidemia detected after culture-confirmed catheter colonization			
Blood culture Group 2	**Catheter, PICC/days before/after IBC**	**BDG (pg/mL) IBC**	**PSEP (pg/mL) IBC**
C. glabrata	*C. glabrata*/3/NC	478	5513
C. parapsilosis	*C. parapsilosis*/3/NC	485	3829
C. glabrata	*C. glabrata*/1/NC	500	1203
C. albicans	*C. albicans*/3/NC	465	1252
C. parapsilosis	*C. parapsilosis*/1/NC	410	927
C. tropicalis	*C. tropicalis*/1/NC	266	2781
C. albicans	*C. tropicalis*/1/NC	457	3222
C. albicans	*C. albicans*/1/NC	511	1715
C. albicans	*C. albicans*/1/NC	514	944
C. glabrata	*C. glabrata*/1/NC	106	1511

**Catheter colonization confirmed 1-3 days after proof of candidemia by culture**			
**Blood culture Group 3**	**Catheter, PICC/days before/after IBC**	**BDG (pg/mL) IBC**	PSEP (pg/mL) IBC
C. albicans	*C. albicans*/NC/2	577	1121
C. tropicalis	*C. tropicalis*/NC/2	1146	2567
C. albicans	*C. albicans*/NC/2	1095	1185
C. albicans	*C. albicans*/NC/2	1115	2581
C. tropicalis	*C. tropicalis*/NC/2	1307	1300
C. lusitaniae	*C. lusitaniae*/NC/3	1251	1292

C. albicans	*C. albicans*/NC/2	1223	4424
C. tropicalis	*C. tropicalis*/NC/2	1133	1972
C. albicans	*C. albicans*/NC/2	1182	10,817
C. albicans	*C. albicans*/NC/2	1270	2325
C. albicans	*C. albicans*/NC/2	1239	3708
C. albicans	*C. albicans*/NC/2	1253	5950

ICB indexed blood culture, **NC** not colonized.

1:Patients without catheter colonization.

2:Candidemia detected after culture-confirmed catheter colonization (Catheter-related candidiasis).

3:Catheter colonization confirmed 1-3 days after proof of candidemia by culture.

Table S4. Monitoring successful treatment of invasive candidiasis using biomarkers.

The concentrations of BDG (*P* = 0.0038) were significantly reduced on day 28; PSEP showed a significant decrease on day 14 (*P* = 0.0012). Biomarker monitoring was performed in patients on days 3, 14, and 28 after initiating echinocandin therapy.

Test_atm_day	n	median	mean	SD	Min	Max	*P-value*
BDG	**17**	**1091**	**1009.8**	**259.45**	**478**	**1307**	
BDG_atm_3	17	1009.5	982.6	305.85	395	1551	
BDG_atm_14	17	1100	916.4	330.09	232	1240	0.2484
BDG_atm_28	17	817	750.3	388.59	84	1342	0.0038
CRP	**17**	**117**	**151.05**	**93.942**	**47**	**338**	
CRP_atm_3	17	142.8	149.8	105.11	23	388	
CRP_atm_14	17	91.5	98.6	58.67	4	189	0.0642
CRP_atm_28	17	86	83.5	62.45	1	212	0.0347
PSEP	**15**	**1786**	**2595.7**	**1713.13**	**837**	**5664**	
PSEP_atm_3	15	1381	2012.8	1565.68	651	5747	
PSEP_atm_14	15	873	1072.3	838.28	305	3100	0.0012
PSEP_atm_28	15	649	602.9	288.62	165	1235	0.0012
PCT	**15**	**2.5**	**13.1**	**25.73**	**0.5**	**100**	
PCT_atm_3	15	1.92	8.8	21.44	0.64	85	
PCT_atm_14	15	1.05	4.6	7.26	0.12	23.3	0.1914
PCT_atm_28	15	0.92	42.1	101.23	0.08	307	0.8647

Wilcoxon signed-rank test - comparison with value on day 3.
atm treatment of invasive candidiasis with echinocandins.
